# Role of Liver CD38 in the Regulation of Metabolic Pathways during Cold-Induced Thermogenesis in Mice

**DOI:** 10.3390/cells11233812

**Published:** 2022-11-28

**Authors:** Andrea Benzi, Sonia Spinelli, Laura Sturla, Markus Heine, Alexander W. Fischer, Friedrich Koch-Nolte, Hans-Willi Mittrücker, Andreas H. Guse, Antonio De Flora, Joerg Heeren, Santina Bruzzone

**Affiliations:** 1 Section of Biochemistry, DIMES, University of Genova, 16132 Genova, Italy; 2 Department of Biochemistry and Molecular Cell Biology, University Medical Center Hamburg-Eppendorf, 20246 Hamburg, Germany; 3 Institute of Immunology, University Medical Center Hamburg-Eppendorf, 20246 Hamburg, Germany

**Keywords:** CD38, NAD(P)(H), hepatic metabolism, thermogenesis, browning

## Abstract

Boosting NAD^+^ levels are considered a promising means to promote healthy aging and ameliorate dysfunctional metabolism. The expression of CD38, the major NAD^+^-consuming enzyme, is downregulated during thermogenesis in both brown and white adipose tissues (BAT and WAT). Moreover, BAT activation and WAT “browning” were enhanced in *Cd38*^−/−^ mice. In this study, the role of CD38 in the liver during thermogenesis was investigated, with the liver being the central organ controlling systemic energy metabolism. Wild-type mice and *Cd38*^−/−^ mice were exposed to cold temperatures, and levels of metabolites and enzymes were measured in the livers and plasma. During cold exposure, CD38 expression was downregulated in the liver, as in BAT and WAT, with a concomitant increase in NAD(H) and a marked decrease in NADPH levels. Glucose-6-phosphate dehydrogenase and the malic enzyme, along with enzymes in the glycolytic pathway, were downregulated, which is in line with glucose-6-P being re-directed towards glucose release. In *Cd38*^−/−^ mice, the cross-regulation between glycolysis and glucose release was lost, although this did not impair the glucose release from glycogen. Glycerol levels were decreased in the liver from *Cd38*^−/−^ animals upon cold exposure, suggesting that glyceroneogenesis, as gluconeogenesis, was not properly activated in the absence of CD38. SIRT3 activity, regulating mitochondrial metabolism, was enhanced by cold exposure, whereas its activity was already high at a warm temperature in *Cd38*^−/−^ mice and was not further increased by the cold. Notably, FGF21 and bile acid release was enhanced in the liver of *Cd38*^−/−^ mice, which might contribute to enhanced BAT activation in *Cd38*^−/−^ mice. These results demonstrate that CD38 inhibition can be suggested as a strategy to boost NAD^+^ and would not negatively affect hepatic functions during thermogenesis.

## 1. Introduction

The liver represents the main metabolic hub of the organism, and its functions are essential for correct metabolic homeostasis. In the liver, different pathways can take place in the fed state, depending on the circulating hormones: glycolysis, fatty acid synthesis, and the release of VLDL. Conversely, in fasted states, the liver generates glucose from glycogen and, through gluconeogenesis, functionally supports other organs. During prolonged fasting, fatty acids are released by adipose tissues and transported in an albumin-bound form to the liver. Here, fatty acids are oxidized and, at least in part, are used for the generation of ketone bodies which, together with released glucose, provide a metabolic fuel for extrahepatic organs and tissues. Aberrant hepatic functions correlate with insulin resistance, type 2 diabetes, obesity, nonalcoholic fatty liver diseases (NAFLD) [1], and its progressive form of non-alcoholic steatohepatitis (NASH). Aged subjects are highly susceptible to NASH and its related complications, such as progressive fibrosis, cirrhosis, and hepatocellular carcinoma [2].

Recently, given the pivotal role of the liver in regulating energy metabolism, the hepatic function has been studied in the context of thermogenesis. Although the brown adipose tissue (BAT) is considered the major site for thermogenesis in humans and other mammals, other tissues, including the liver and skeletal muscle, contribute to cold adaptation. Cold exposure increases hepatic gluconeogenesis, total liver and mitochondrial mass, the respiration capacity of hepatocytes, and liver temperature [3,4]. Furthermore, the liver influences thermogenesis by producing and releasing factors that act as metabolic boosters, such as bile acids (BAs) [5,6], acyl-carnitines (captured exclusively by BAT and boosting heat production, ref. [4]), and fibroblast growth factor 21 (FGF21). FGF21 is produced and released by the liver during thermogenesis, as well as in response to prolonged fasting, re-feeding, and macronutrient imbalance. It might affect the sympathetic nervous system and, thus, indirectly promotes adipose tissue thermogenic activation [7].

Nicotinamide adenine dinucleotide (NAD^+^, with its phosphorylated form NADP^+^) is one of the most important cofactors in living cells. Both NAD^+^ and NADP^+^ take part in several redox reactions, including its reduction in the nicotinamide (NAM) ring to generate NADH and NADPH, respectively. A specific NAD^+^/NADH ratio is essential for cellular metabolism and mitochondrial function, whereas the NADPH/NADP^+^ ratio is a sensor of the cellular antioxidant potential since NADPH is involved in detoxification from reactive oxygen species [8].

Besides being a coenzyme for dehydrogenases, NAD^+^ is also a substrate of NAD^+^-consuming enzymes, which affect several cellular processes by regulating gene expression, protein localization, activity, and calcium homeostasis. There are three main enzyme families able to metabolize NAD^+^, all hydrolyzing the bond between the NAM ring and ribose: Sirtuins (SIRTs), NAD^+^-ases/ADP-ribosyl cyclases, mono ADP-ribosyl transferases and poly ADP-ribose polymerases (PARPs) [9,10,11]. SIRTs are NAD^+^-dependent deac(et)ylases, which regulate gene expression and protein activities. PARPs consume NAD^+^ and, by removing NAM, link different ADP-ribose (ADPR) moieties to a target protein, influencing its activity. CD38 is the main enzyme endowed with NAD^+^-ase activity. CD38 is a transmembrane enzyme, expressed by several cell types, which produce ADPR and cyclic ADPR (cADPR), two Ca^2+^-mobilizing second messengers [12]. It has recently been reported that CD38 can also carry-out the conversion of 2′-deoxy-NAD^+^ to 2′-deoxy-ADPR (2dADPR) [13].

CD38 pharmacological inhibition has been largely studied in hematological malignancies, and different anti-CD38 monoclonal antibodies are currently in use for the treatment of multiple myeloma [14,15]. Nevertheless, CD38 inhibition is also being considered a promising approach to treat several pathological conditions, including, but not limited to, allergic airway diseases [16], cardiovascular diseases [17], vascular thrombosis, disordered inflammation, and aberrant immune reactivity [18], obesity-associated metabolic abnormalities [19,20] and Duchenne muscular dystrophy [21].

In these pathological contexts, the beneficial effect obtained by CD38 inhibition is due to the fact that CD38 represents the major NAD^+^ degrading activity in different tissues; therefore, CD38 inhibition determines a boost of intracellular NAD^+^ levels [22]. Indeed, CD38 inhibition is also considered one of the most promising targets for the treatment of age-related disorders when NAD^+^ declines in different organs, including the liver, leading to organ dysfunction [20,23,24,25]. Thus, CD38 absence protects from NAFLD and hepatic steatosis induced by a high-fat diet (HFD) as a consequence of SIRTs activation fostered by the higher NAD^+^ levels [26]. Indeed, during aging and/or overfeeding, NAD^+^ levels decline as a consequence of a reduced synthesis and/or overexpression of degraded pathways. Nicotinamide phosphoribosyl transferase (NAMPT) is downregulated in the liver of HFD-fed mice [23], leading to low NAD^+^ levels in the organ [24]. Furthermore, senescent cell-derived factors induce a pro-inflammatory commitment in the immune cells infiltrating from the hepatic tissue, leading to CD38 overexpression and consequent NAD^+^ reduction [27]. Overall, different findings indicate that the detrimental role of CD38 in the liver is associated with its NAD^+^-consuming activity.

Besides being inversely related to NAD^+^ levels, CD38 has been studied in liver-associated disorders as an enzyme involved in the regulation of Ca^2+^ homeostasis. For instance, CD38 plays a role in promoting the activation of hepatic stellate cells that are responsible for extracellular matrix protein production and the induction of hepatic fibrosis, by producing Ca^2+^-mobilizing molecules [28].

Finally, regarding CD38 and thermogenesis, a recent study demonstrated that CD38 expression is downregulated in both brown and white adipose tissues upon cold exposure, with a consequent increase in NAD^+^ and NADP(H) levels, respectively [29,30]. Given the important role of CD38 in physio-pathological conditions in the liver, we investigated its role in this organ during cold-induced thermogenesis.

## 2. Materials and Methods

### 2.1. In Vivo Experiments

All in vivo experiments were conducted in accordance with the laws and institutional guidelines for animal care and were performed with the permission of the Animal Welfare Officers at University Medical Center Hamburg-Eppendorf. The mice were housed in a temperature- and light-controlled conditions (12 h light cycle) with food and water ad libitum. *Cd38*^−/−^ mice were kindly provided by Prof Francis Lund (The University of Alabama at Birmingham, Birmingham, AL, USA). Three months old wild type (WT) and *Cd38*^−/−^ mice were used for this study (7–8 animals/group). Two groups (the knockout mice and WT mice, used as controls) were kept for 7 days at 30 °C; the other animals were kept at 22 °C, and two groups were kept for the last 24 h at 6 °C. The mice fasted during the last 4 h before the end of the experiments. The mice were euthanized, and interscapular BAT (iBAT) was collected, together with inguinal WAT (iWAT) and liver, and flash frozen in liquid nitrogen for future investigations. Blood was also collected, centrifuged, and plasma was frozen.

### 2.2. Measurement of NAD(P)(H) Content

NAD(P)(H) levels were evaluated in the liver of WT and *Cd38*^−/−^ mice. Before their disruption with 0.6 M HClO_4_ (PCA) or 0.1 M NaOH, samples were weighed to normalize the dinucleotide content. All samples were sonicated and centrifuged at 15,000× *g* for 10 min PCA. The samples were neutralized by diluting the extracts in 100 mM sodium phosphate buffer (pH 8) and were submitted to assays of NAD^+^ and NADP^+^ content; the samples in NaOH were warmed at 72 °C for 10 min, neutralized in 10 mM Tris-HCl, pH 6, and used for NADH and NADPH determination. NAD(P)(H) contents were assessed with a specific enzyme cyclic assay [31].

### 2.3. Enzymatic Assays

The livers were cut into small pieces and homogenized by sonication in lysis buffer (0.01 M Na_2_HPO_4_/NaH_2_PO_4_, pH 7.4) in the presence of protease inhibitors, and the lysate protein content was determined by the Bradford method. All assays of the different enzymatic activities were carried out using 25–50 μg of protein for each lysate.

#### 2.3.1. NAD^+^-Ase

NAD^+^-ase activity was estimated by adding 0.4 mM NAD^+^ as a substrate to the incubation mixture (20 mM Tris-HCl, pH 7, 200 μL). Aliquots of the incubations were withdrawn at 0, 3, and 10 min: the ADPR content was evaluated by HPLC analysis [32].

#### 2.3.2. NAD^+^ Synthesis

NAD^+^ synthesis starting from NAM, 5-phosphoribosyl 1-pyrophosphate (PRPP), and ATP was evaluated as in [29]. Briefly, the different lysates were added to 100 μL reaction mixtures (3 mM ATP, 5 mM MgCl_2_, 0.5 mM PRPP, 2.5 mM NAM, 50 mM Tris-HCl, pH 7.4) and incubated at 37 °C. Aliquots were withdrawn at 0, 45, and 90 min, and the reaction was stopped by the addition of PCA (0.6 M final concentration). The NAD^+^ content was measured by enzymatic cycling assay as described above.

#### 2.3.3. NMNAT Activity

NMNAT activity was evaluated by incubating each lysate in the presence of 0.5 mM Nicotinamide mononucleotide (NMN), 3 mM ATP, 5 mM MgCl_2_, 50 mM Tris-HCl, pH 7.4. Aliquots were withdrawn at 0, 30, and 60 min. PCA (0.6 M final concentration) was added, and NAD^+^ content was evaluated by the enzymatic cycling assay, as above.

#### 2.3.4. NAD^+^ Kinase Activity

NAD^+^ kinase activity was measured by incubating each lysate in the presence of 1 mM NAD^+^, 10 mM ATP, 2 mM NAM, and 10 mM MgCl_2_ in 100 mM Tris-HCl, pH 7.8. Aliquots were withdrawn at 0, 30, and 90 min, the reactions were stopped by adding PCA (0.6 M final concentration), and the NADP^+^ content was evaluated using the cycling assay (see above).

#### 2.3.5. LDH Activity

LDH (lactate dehydrogenase) activity was measured using 50 or 25 ug of protein for each lysate by incubating them in the presence of L-(+)-Lactate (10 mM) and NAD^+^ (3 mM) in Tris-HCl (40 mM final concentration, pH 8). The activity was measured following the reduction in NAD^+^ to NADH, as detected by a fluorescence microplate reader (Em: 460 nm, and Ex: 350 nm, FLUOstar OPTIMA, BMG LabTech, Offenburg, Germany), at 25 °C.

#### 2.3.6. Glucose-6 Phosphate Dehydrogenase Activity

Approximately 20 mg of each organ were lysed in an ice-cold buffer [25 mM Tris-HCl (pH 7.4), 1 mM EDTA, and protease inhibitors] by brief sonication. The lysates were centrifuged at 10,000× *g* for 10 min at 4 °C, and the supernatants were collected. G6PD activity was evaluated on 25 µg of protein for each supernatant by measuring the reduction in NADP^+^ in the reaction buffer [100 mM Tris-HCl (pH 7.4), 0.5 mM EDTA, 10 mM MgCl_2_, 0.2 mM NADP^+^, and 0.6 mM G6P] at 25 °C as in [31].

### 2.4. Glucose-6 Phosphate Content

Livers from WT and *Cd38*^−/−^ mice were weighed and then disrupted in 0.6 M PCA. After a brief sonication, the samples were centrifuged at 15,000× *g* for 10 min at 4 °C. The glucose-6 phosphate (G6P) content was evaluated by the enzymatic cycling assay used to determine NADP(H) levels, but with G6P limiting the metabolite to be measured. The assay was carried out upon the addition of 0.2 mM NADP^+^, 0.02 U/mL G6PD, 20 μM resazurin, 5 μg/mL diaphorase, 10 μM FMN, 10 mM NAM, and 100 mM sodium phosphate, pH 8.0: the endogenous G6P (present in the liver extracts was utilized by the added G6PD. Resofurin-derived fluorescence was measured in a microplate reader (Fluostar Optima, BMG Labtechnologies GmbH, Ortenberg, Germany) as in the NADP(H) evaluation assay (see above). A standard curve of G6P (ranging from 250 to 0.4 µM) was run in parallel.

### 2.5. Western Blot Analysis

The liver was lysed in lysis buffer (1 mM EDTA, 150 mM NaCl, 1 mM FH_4_N, 10 mM NAM, 0.5 mM DTT, 0.01 mM Trichostatin A) in the presence of protease inhibitors using a tissue lyser. The homogenates were centrifuged. Lysates (20 µg proteins) were loaded on a 10% polyacrylamide gel, and the proteins were separated by SDS-PAGE and transferred to nitrocellulose membranes. Detection was performed with primary antibodies (see below), following incubation with the appropriate secondary antibodies and ECL detection (GE Healthcare, Milan, Italy). Band intensity was quantified with the ChemiDoc imaging system (Bio-Rad, Milan, Italy).

The following primary antibodies were used: anti-CD38 (kindly provided by Prof. Fabio Malavasi, University of Torino, Turin, Italy); anti-vinculin (Cell Signaling Technology, #4650 Danvers, MA, USA); anti-SOD2/MnSOD (acetyl K68) antibody (Abcam, ab137037, Boston, MA, USA); anti-SOD2/MnSOD (Abcam, ab13533); anti-GAPDH (Merck Millipore, G8795, Milano, Italy).

### 2.6. Glycogen

Glycogen levels were evaluated using a Glycogen assay kit (Cayman, 700480, Ann Arbor, MI, USA).

### 2.7. Bile Acids, Cholesterol, Triglyceride and Glycerol Measurements 

The total triglyceride (TGs) and glycerol levels were measured in homogenized livers using the Triglycerides quantification kit (Abcam, Ab65336). Triglyceride and cholesterol levels in the plasma were evaluated with commercial kits (Roche, Basel, Switzerland). The total BA levels in the plasma were measured using the Bile Acid Assay Kit (Merck Millipore, MAK309).

### 2.8. qPCR Analyses

RNA was isolated from the liver using peqGOLD TriFast (Peqlab, Erlangen, Germany) by homogenizing the tissues with TissueLyser (Qiagen, Venlo, The Netherland) and purifying RNA with the NucleoSpin RNAII Kit (Macherey-Nagel, Allentown, PA, USA). Afterwards, cDNA was prepared with a High-Capacity cDNA Archive Kit (Applied Biosystems, Waltham, MA, USA). The cDNA was used as a template for qPCR analysis: reactions were performed in an iQ5 real-time PCR detection system (Bio-Rad) following the experimental conditions described before [33]. PCR primers were designed through Prime3 (v. 0.4.0) online software and are listed in Table 1. Statistical analysis of the qPCR was performed using the iQ5 Optical System Software version 1.0 (Bio-Rad) based on the 2^−ΔΔCt^ method [34]. The dissociation curve for each amplification was analyzed to confirm the absence of unspecific PCR products.

### 2.9. Statistical Analyses

The groups were compared by an unpaired Student’s *t*-test or by one-way ANOVA followed by Tukey’s Test, using GraphPad software. Values of *p* < 0.05 were considered significant.

## 3. Results

### 3.1. NAD(H) and NADP(H) Levels Are Modified in Liver during Cold Exposure

**Figure 1:**
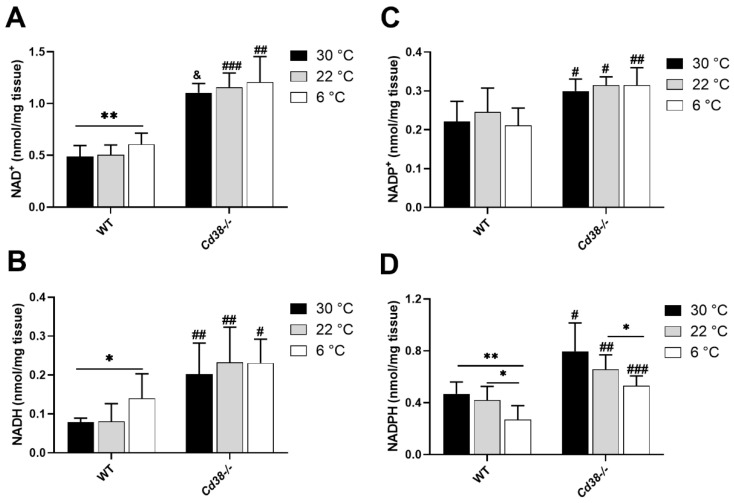
Cold exposure increases NAD(H) levels and decreases NADPH levels in liver from WT mice. Livers were harvested from WT and *Cd38*^−/−^ mice housed at 30 °C for 7 days (30 °C, black bars), or at 22 °C for 7 days (22 °C, grey bars), or at 22 °C for 6 days and at 6 °C for 1 day (6 °C, white bars). For dinucleotide content assay, tissues were minced on ice and then deproteinized in PCA or NaOH. Acid-extracted samples were used to evaluate NAD^+^ and NADP^+^ (**A**,**C**), whereas base-extracted samples were used to determine NADH and NADPH levels (**B**,**D**). Results are the means ± SD (*n* = 6–7). Data analyzed by ANOVA with Tukey’s test: *, *p* < 0.05; **, *p* < 0.01; data analyzed by Student’s *t*-test: ^#^, *p* < 0.05; ^##^, *p* < 0.01; ^###^, *p* < 0.001; ^&^, *p* < 0.00001 compared with the corresponding WT.

Cold exposure determined the increase in levels of NAD(H) in BAT and of NADP(H) in WAT in mice [29]. Here, we evaluated the impact of this stimulus on NAD(P)(H) in the liver, the main metabolic hub in the body. NAD(H) levels were measured by an enzymatic cycling assay on the liver of WT and *Cd38*^−/−^ mice either exposed to the cold or housed at 30 °C or 22 °C: both NAD^+^ and NADH levels were increased in the cold-exposed mice (by 1.3 and 1.8 folds, respectively), compared to mice kept at neutral temperatures (Figure 1A,B).

In line with results obtained in the adipose tissue, the livers harvested from *Cd38*^−/−^ mice exhibited remarkably high levels of NAD(H) in comparison with WT mice (approximately 2.5–3-fold higher for both NAD^+^ and NADH at all temperatures; Figure 1A,B), suggesting that CD38 is a major NAD^+^-consuming enzyme which can also be found in liver. Accordingly, no significant changes in NAD(H) content were detected in the liver of *Cd38*^−/−^ mice at the three different housing temperatures.

Next, NADP^+^ and NADPH were measured in the liver, harvested from animals kept at 30 °C, 22 °C, or 6 °C. As evaluated by the specific enzymatic cycling assay, NADP^+^ levels were not modified in the liver of mice exposed to cold temperatures, either in WT or in *Cd38*^−/−^ (Figure 1C). On the other hand, NADPH levels in the liver were strongly decreased by cold exposure. In WT mice, cold exposure resulted in a 45% decline of NADPH, in comparison with 30 °C-housed mice (Figure 1D). Similarly, in the liver of *Cd38*^−/−^ mice, cold exposure caused a reduction in NADPH levels by approximately 35% in comparison with 30 °C-housed mice (Figure 1D). NADP(H) levels in *Cd38*^−/−^ were significantly higher than in WT mice (Figure 1C,D), indicating that CD38 activity affects the amount of the NADP(H) pool, along with the NAD(H) one. Increased NADPH levels in the liver of *Cd38*^−/−^ mice may be important to sustain lipid synthesis (see below).

### 3.2. CD38 Expression Is Downregulated in Liver upon Cold Exposure

**Figure 2:**
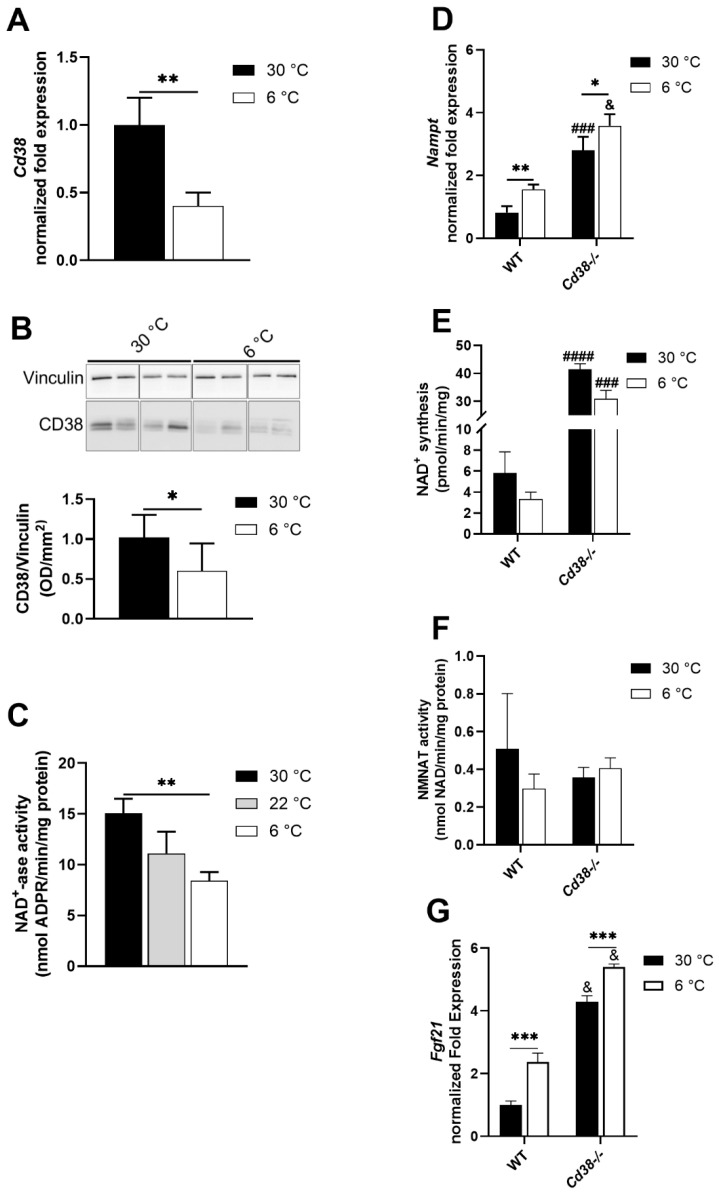
Cold exposure down-regulates CD38 levels in liver from WT mice. Livers were harvested from WT and *Cd38*^−/−^ mice housed at 30 °C for 7 days (30 °C, black bars), or at 22 °C for 7 days (22 °C, grey bars), or at 22 °C for 6 days and at 6 °C for 1 day (6 °C, white bars). (**A**–**C**), CD38 expression was measured by RT-PCR (**A**), Western blot analyses (**B**), and by evaluating the enzymatic activity and measuring ADPR production starting from NAD^+^ (**C**). (**D**,**G**) *Nampt* and *Fgf21* gene expression levels were evaluated by RT-PCR. (**E**,**F**), NAD^+^ synthesis was evaluated measuring NAD^+^ production starting from NAM, PRPP, and ATP (**E**), or starting from NMN and ATP (**F**). Results are the means ± SD (*n* = 4–5). Data analyzed by ANOVA with Tukey’s test: *, *p* < 0.05; **, *p* < 0.01; ***, *p* < 0.001; data analyzed by Student’s *t*-test: ^###^, *p* < 0.001; ^####^, *p* < 0.0001; ^&^, *p* < 0.00001, compared with the corresponding WT.

Cold exposure determined a significant decrease in the CD38 expression in the liver, as measured using three different approaches: RT-PCR analysis (by approximately 60%, Figure 2A), Western blot analysis (by approximately 40%, Figure 2B), and an assay of NAD^+^ ase activity (by 45%, Figure 2C). Thus, cold exposure determined the downregulation of CD38 expression, as observed in BAT and WAT [29], and could account, at least in part, for the corresponding NAD(H) increase at cold temperatures.

### 3.3. NAMPT and FGF21 Are Upregulated in Liver upon Cold Exposure

To identify other possible explanations for the increased NAD(H) levels observed in the liver upon cold exposure, we verified whether the NAD^+^-synthetizing activity from NAM was upregulated. NAMPT is the main enzyme in the salvage pathway, converting NAM to NMN: *Nampt* mRNA levels were significantly higher in both WT and *Cd38*^−/−^ mice exposed to the cold, in comparison with mice housed at 30 °C (Figure 2D). However, cold exposure did not modulate the total NAD^+^-synthesizing activity, starting from NAM and PRPP and producing NAD^+^ by utilizing ATP (Figure 2E). Notably, basal (30 °C) levels of both the Nampt expression and NAD^+^ synthesizing activity were strongly increased in *Cd38*^−/−^ mice compared to WT (Figure 2D,E). Conversely, the NMNAT enzymatic activity (converting the NAMPT-produced NMN to NAD^+^, with ATP consumption) was not significantly modified in the different conditions, demonstrating that the cold stimulus did not influence this enzymatic activity (Figure 2F). Recently, Higgins and colleagues unveiled an interaction occurring between NAMPT and SIRT1 that promoted FGF21 production [35]. Thus, *Fgf21* mRNA levels were evaluated: as expected, liver *Fgf21* mRNA levels were increased in cold-exposed WT mice (Figure 2G), in line with previous reports [36]. Interestingly, *Fgf21* mRNA levels were strongly up-regulated in *Cd38*^−/−^ mice, both during cold acclimatization and in the control condition, with cold exposure, further increasing *Fgf21* production (Figure 2G).

### 3.4. Downregulated Expression of G6PD and Malic Enzyme Are Responsible for the Decrease in NADPH in Liver of WT and Cd38^−/−^ Mice upon Cold Stimulation

**Figure 3:**
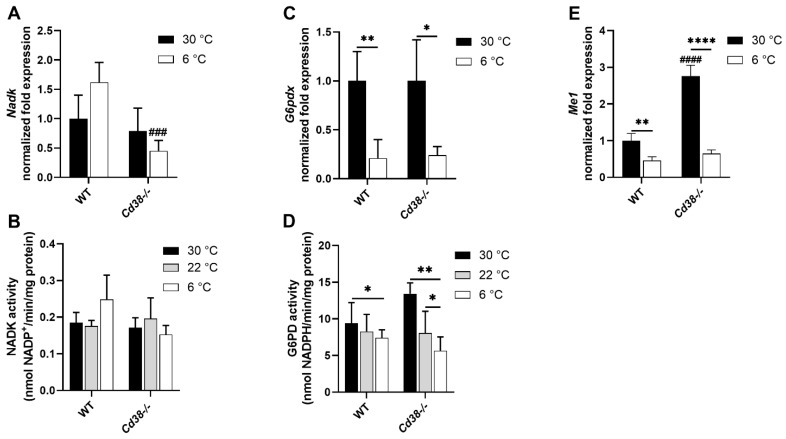
G6PD and malic enzyme are downregulated in liver of WT and *Cd38*^−/−^ mice. Livers were harvested from WT and *Cd38*^−/−^ mice housed at 30 °C for 7 days (30 °C, black bars), or at 22 °C for 7 days (22 °C, grey bars), or at 22 °C for 6 days and at 6 °C for 1 day (6 °C, white bars). *Nadk*, *G6pdx* and *Me1* expressions were measured by RT-PCR (**A**,**C**,**E**). NADK activity was evaluated by measuring the NADP^+^ production, starting from NAD^+^ and ATP (**B**). G6PD activity was evaluated by measuring NADPH production, starting from G6P and NADP^+^ (**D**). Results are the means ± SD (*n* = 4–5). Data analyzed by ANOVA with Tukey’s test: *, *p* < 0.05; **, *p* < 0.01; ****, *p* < 0.0001; data analyzed by Student’s *t*-test: ^###^, *p* < 0.001; ^####^, *p* < 0.0001 compared with the corresponding WT.

To investigate the mechanisms leading to the NADPH decrease in the liver during thermogenesis, the expression levels of NAD^+^ kinase, G6PD, and malic enzyme (ME) were evaluated. NAD^+^ kinase is responsible for NADP^+^ production starting from NAD^+^; G6PD and malic enzyme are the main enzymes involved in NADP^+^ reduction to NADPH. NADK gene expression and enzymatic activity were slightly, yet not significantly, increased in the liver of WT mice upon cold exposure (Figure 3A,B). *Cd38*^−/−^ mice did not exhibit an up-regulation of the *Nadk* gene and activity upon exposure to cold (Figure 3A,B).

G6PD expression and activity were remarkably downregulated upon cold exposure in both WT and *Cd38*^−/−^ mice, compared with the mice housed at a controlled temperature (Figure 3C,D). Concerning the malic enzyme, *Cd38*^−/−^ mice displayed a significantly higher *Me1* expression in comparison with WT mice (Figure 3E). However, *Me1* was greatly downregulated upon cold exposure in both WT and *Cd38*^−/−^ mice, compared with mice housed at the control temperature (Figure 3E).

Altogether, the results from the G6PD and malic enzyme expression are in line with the observed decrease in NADPH content in the liver and suggest that the entrance of hepatic G6P in the pentose phosphate pathway is reduced during thermogenesis in both WT and *Cd38*^−/−^ mice, this decrease being stronger in the latter animals.

### 3.5. Glycolytic and Gluconeogenic Pathways Are Not Affected by Cold Exposure in Cd38^−/−^ Mice

**Figure 4:**
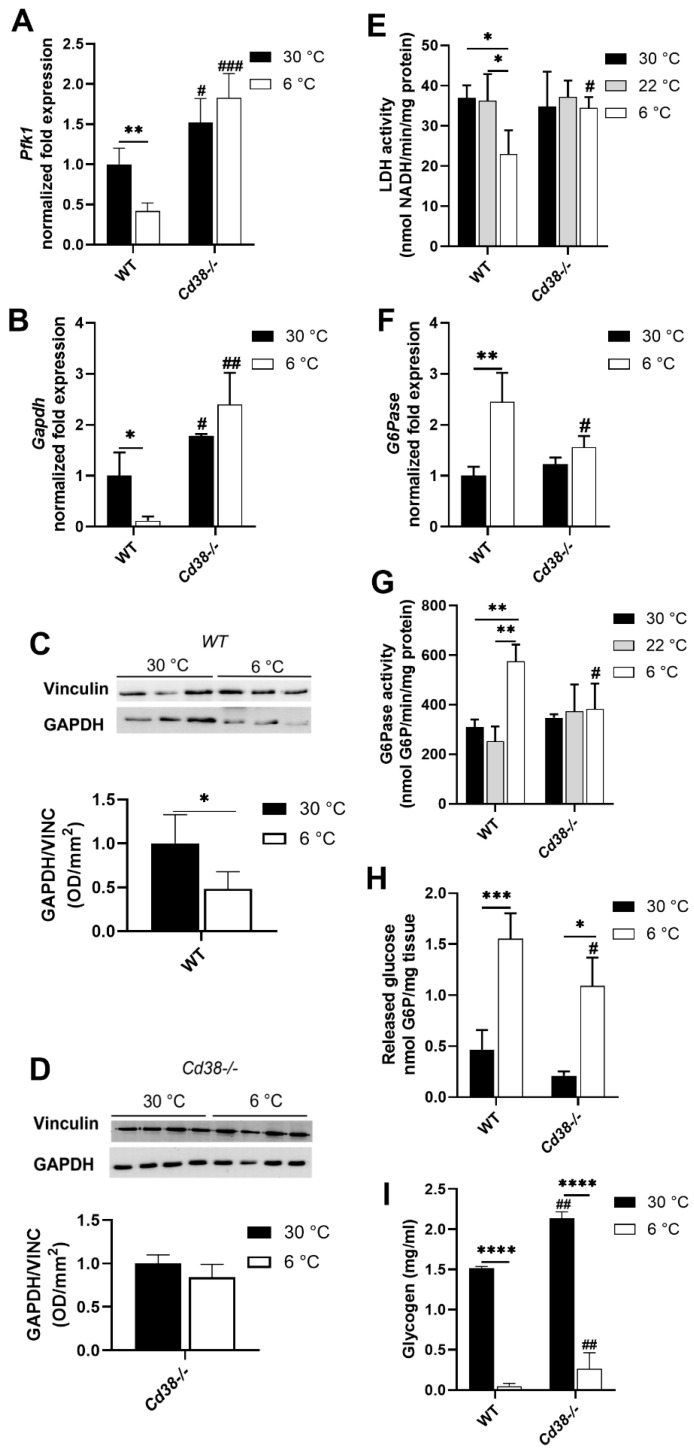
Cold exposure determines a metabolic switch from glycolysis to gluconeogenesis in WT mice but not in mice lacking *Cd38*^−/−^. Livers collected from WT and *Cd38*^−/−^ mice housed at 30 °C for 7 days (30 °C, black bars), or at 22 °C for 7 days (22 °C, grey bars), or at 22 °C for 6 days and at 6 °C for 1 day (6 °C, white bars) were analyzed for gene expression and enzymatic activity quantification. *Pfk1*, *Gapdh*, and *G6pase* gene expression levels were measured by RT-PCR (**A**,**B**,**F**). GAPDH protein expression levels were measured by Western blot analyses (**C**,**D**). LDH activity was measured by quantifying NADH-produced incubating liver lysates with NAD^+^ and L(+)-lactate (**E**). G6Pase activity was evaluated as G6P depletion from the incubation with liver lysates (**G**). G6P levels were measured in acid-extracted samples, using a cycling assay (**H**). Hepatic glycogen was measured in fresh minced tissues by glucose quantification, upon glycogen hydrolysis, using a commercially available kit (**I**). All results are the means ± SD of 4–5 independent determinations for each condition. Data analyzed by ANOVA with Tukey’s test: *, *p* < 0.05; **, *p* < 0.01; ***, *p* < 0.001; ****, *p* < 0.0001; data analyzed by Student’s *t*-test: ^#^, *p* < 0.05; ^##^, *p* < 0.01; ^###^, *p* < 0.001 compared with the corresponding WT.

To evaluate the fate of hepatic G6P in the liver during thermogenesis in WT and *Cd38*^−/−^ mice, the expression of enzymes involved in the main glucose pathways was analyzed. *Pfk1* (phosphofructokinase 1) and *Gapdh* (glyceraldhyde-3-phosphate dehydrogenase) expression and LDH activity were tested to estimate the glycolytic pathway rate in WT and *Cd38*^−/−^ mice. *Pfk1* and *Gapdh* expression levels were significantly lower in the liver of WT mice exposed to the cold than in mice housed at 30 °C (by approximately 2.5 and 8 folds, respectively). Conversely, this downregulation was abrogated in *Cd38*^−/−^ mice, in which even a trend to increased *Pfk1* and *Gapdh* levels was detected in the cold-exposed mice (Figure 4A,B). Western blot analyses confirmed that hepatic GAPDH expression was downregulated (by approximately 50%) in WT but not in *Cd38*^−/−^ mice exposed to the cold (Figure 4C,D). In line with this, a marked reduction in LDH enzymatic activity was detected in the liver from WT but not from *Cd38*^−/−^ mice exposed to cold temperatures, compared with mice housed at 22 °C and 30 °C (Figure 4E).

Next, the gene expression and activity of the gluconeogenic enzyme G6Pase (glucose 6-phosphatase) were evaluated. As shown in Figure 4F,G, cold exposure determined a marked increase in G6Pase expression and activity (approximately 2.5 and 2 folds, respectively) in WT mice. Conversely, the cold stimulus did not affect G6Pase expression and activity in the liver of *Cd38*^−/−^ mice (Figure 4F,G). Hepatic G6P levels were also evaluated: in line with what was observed for G6Pase, the amount of released glucose was higher in WT than in *Cd38*^−/−^ mice upon cold exposure (Figure 4H). Glycogen levels were higher in the *Cd38*^−/−^ mice housed at 30 °C (Figure 4I) and in line with observations reported by [37]. Interestingly, they were drastically decreased in both WT and *Cd38*^−/−^ mice exposed to the cold (Figure 4I). The remaining glycogen was significantly higher in the liver of cold-exposed *Cd38*^−/−^ mice than in WT mice (Figure 4I).

Altogether, these data suggest that upon cold exposure, the liver shifts its metabolism from glycolysis to gluconeogenesis/glycogenolysis and that CD38 plays a role in this cross-regulation.

### 3.6. CD38 Deletion Affects Pdh Expression and SIRT3 Enzymatic Activity in Liver

**Figure 5:**
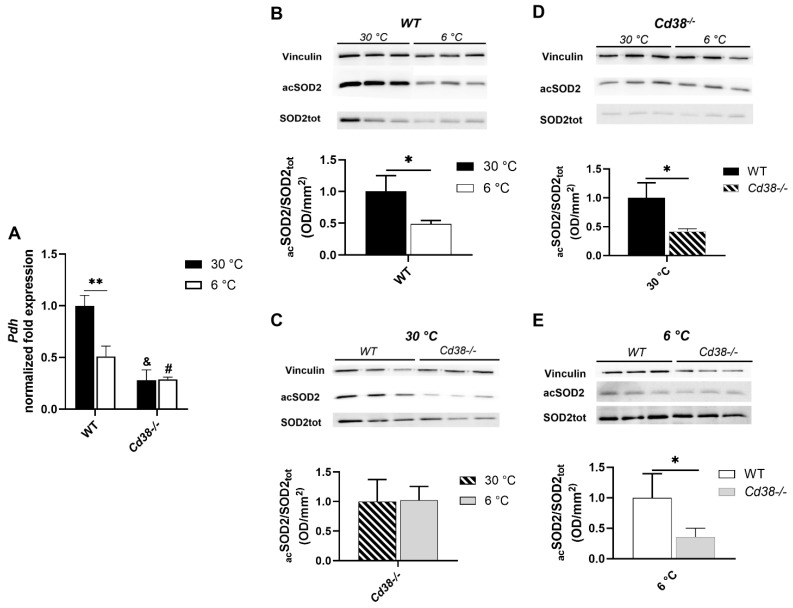
Cold exposure SIRT3 activity in WT, but not in *Cd38*^−/−^ mice, with SIRT3 over-activated at warm temperatures in *Cd38*^−/−^ mice. Livers from WT and *Cd38*^−/−^ mice, kept for 1 week at 30 °C (black bars) or 6 days at 22 °C followed by 1 day at 6 °C (white bars), were used. *Pdh* expression was evaluated by RT-PCR (**A**). SIRT3 activity was evaluated by Western blot, measuring the acetylation level of SOD2, a substrate of SIRT3 activity (**B**–**E**). Results of quantification are the mean ± SD from *n* = 3 animals for each condition. Data analyzed by ANOVA with Tukey’s test: *, *p* < 0.05; **, *p* < 0.01; data analyzed by Student’s *t*-test: ^#^, *p* < 0.05; ^&^, *p* < 0.00001 compared with the corresponding WT.

Given the differences in glucose metabolisms occurring in WT and *Cd38*^−/−^ mice during thermogenesis (Figure 4), pyruvate dehydrogenase (*Pdh*) expression was evaluated by RT-PCR. Upon cold exposure, *Pdh* expression was significantly reduced in WT but not in *Cd38*^−/−^ mice (Figure 5A). Thus, as for the enzymes in the glycolytic pathway, the regulation of *Pdh* expression was lost in the absence of CD38. *Pdh* levels were significantly reduced in the liver from *Cd38*^−/−^ mice compared to WT mice (Figure 5A), in line with a previous report [38].

Since SIRT3 (a member of the sirtuin family) is a master regulator of mitochondrial metabolism known to be activated during thermogenesis [39], SIRT3 activity was compared in the different conditions by measuring the acetylation levels of SOD2 (superoxide dismutase 2, one of its known substrates), as revealed by the Western blot analysis using a specific antibody against the acetylated form, normalized to the total SOD2 expression. The higher the value (i.e., the more SOD2 is acetylated), the lower SIRT3 activity is. As shown in Figure 5B, SOD2 acetylation levels were lower (by approximately 50%) when WT mice were exposed to the cold, confirming that SIRT3 was activated during cold exposure in WT mice. Conversely, in the liver of *Cd38*^−/−^ mice, SIRT3 activity was not enhanced by cold exposure (Figure 5C). In the mice housed at both 30 °C and 6 °C, SIRT3 was more active in the liver from *Cd38*^−/−^ than in the liver from WT mice, as if it had already reached the highest achievable activity and was not further potentiated by cold exposure (Figure 5C–E). Thus, *Cd38*^−/−^ mice displayed higher SIRT3 activity in the liver, at both temperatures, in comparison with the liver from WT mice, suggesting that CD38 (likely via the modulation of NAD^+^ levels) is crucial for SIRT3 regulation, in line with [38].

### 3.7. Cd38 Deletion Promotes Hepatic Lipid Synthesis and Release at Warm Temperature and BAs Release at Cold Temperature

**Figure 6:**
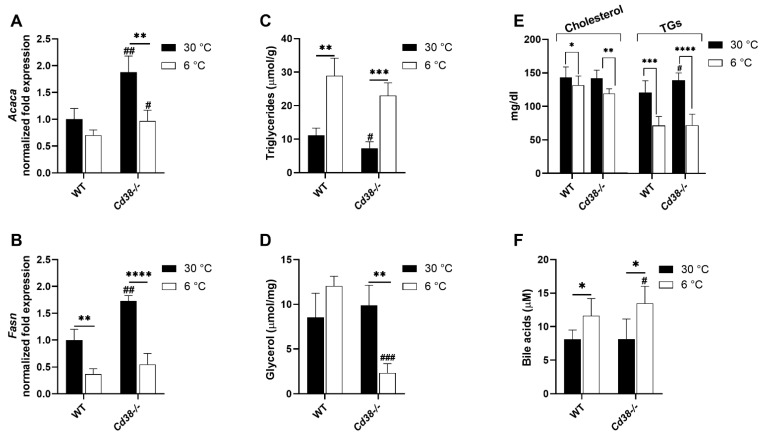
Changes in lipid metabolism induced by cold in WT and *Cd38*^−/−^ mice. Livers collected from WT and *Cd38*^−/−^ mice housed at 30 °C for 7 days (30 °C, black bars), or at 22 °C for 7 days (22 °C, grey bars), or at 22 °C for 6 days and at 6 °C for 1 day (6 °C, white bars) were analyzed for gene expression. *Acaca* and *Fasn* expression levels were measured by RT-PCR (**A**,**B**). Total TGs and free glycerol were detected in freshly chopped livers by quantitative fluorometric assay, using a commercially available kit (**C**,**D**). Blood samples from each mouse were spun down and the resulting serum were used to quantify TGs, cholesterol, and BAs (**E**,**F**). Results are the mean ± SD (*n* = 4–5). Data analyzed by ANOVA with Tukey’s test: *, *p* < 0.05, **, *p* < 0.01, ***, *p* < 0.001, ****, *p* < 0.0001; data analyzed by Student’s *t*-test: ^#^, *p* < 0.05, ^##^, *p* < 0.01, ^###^, *p* < 0.001, compared with the corresponding WT.

Finally, hepatic lipid metabolism was compared in the two mice strains at different temperatures. First of all, the gene expression of the fatty acid synthesis enzymes *Acaca* (acetyl CoA carboxylase) and *Fasn* (fatty acid synthase) were evaluated. *Acaca* gene expression levels showed only a reduced trend, whereas the expression of *Fasn* was significantly decreased (by approximately 2.5 folds) in cold-exposed WT mice, in comparison with WT mice housed at 30 °C (Figure 6A,B). Interestingly, the expression of *Acaca* and *Fasn* was higher in *Cd38*^−/−^ mice than in WT mice at 30 °C (by approximately 1.9 and 1.75 folds, respectively). An increased fatty acid synthesis in *Cd38*^−/−^ mice might be sustained by increased NADPH levels (Figure 1D). However, *Acaca* and *Fasn* expression levels were significantly downregulated (by approximately 2 and 3 folds for *Acaca* and *Fasn*, respectively), reaching levels similar to those in WT mice at 6 °C (Figure 6A,B). Despite the higher expression of enzymes involved in the fatty acid synthesis, *Cd38*^−/−^ mice had a reduced content of hepatic TGs compared to WT mice when housed at 30 °C (Figure 6C). Instead, the plasma TG levels were increased in *Cd38*^−/−^ mice (Figure 6E), suggesting that TG release is enhanced at warm temperatures in the absence of CD38.

In line with liver assembling, the levels of TGs, in order to be exported to the other organs in VLDL during thermogenesis, were greatly increased during cold exposure, both in WT and in *Cd38*^−/−^ mice (Figure 6C). Interestingly, glycerol levels were dramatically decreased in the liver from *Cd38*^−/−^ animals upon cold exposure, suggesting that glyceroneogenesis, as gluconeogenesis, is not properly activated in the absence of CD38 during thermogenesis (Figure 6D).

As mentioned, serum TGs alongside cholesterol, were lower both in the WT and *Cd38*^−/−^ mice housed at 6 °C in comparison to the respective control. Moreover, the BAs concentration was measured in the serum, as the BAs are considered one of the factors influencing thermogenesis (see Introduction). As expected, the BAs levels increased upon cold exposure in WT mice. Importantly, BA increase was higher in the serum of *Cd38*^−/−^ cold-exposed mice in comparison to the WT mice exposed at cold temperatures (Figure 6F). These results, together with the increased FGF21 synthesis, are in line with the enhanced WAT browning and BAT activation observed in *Cd38*^−/−^ mice [29,30].

## 4. Discussion

CD38 expression is downregulated in both BAT and WAT during thermogenesis [29]. In the present study, we demonstrated that, besides the adipose tissue, the down-regulation of CD38 also occurred in the liver upon cold exposure (Figure 2A–C). Differently from WAT and BAT, CD38 downregulation in the liver of WT mice determined not only an increment in NAD^+^ but also in NADH levels (Figure 1A,B). This finding suggests that in the liver, when an enhancement in energy expenditure occurs, NAD^+^ saved through CD38 suppression is rapidly reduced. As a consequence, the NAD^+^/NADH ratio decreases upon cold exposure. In line with our result, Wei et al. observed that cold exposure causes the increase in both NAD^+^ and NADH levels in the liver, with a strong statistical relevance on day 2 of cold acclimation [40]. However, their study did not consider CD38 as the underlying mechanism explaining the increment in NAD(H) levels; rather, the authors focused on an increased NAD^+^ synthesis: NAMPT and NMNAT expression was unchanged after 24 h of cold exposure, as verified at the mRNA and protein levels, but was significantly upregulated after 48 h. In our study, an increase in *Nampt* expression in the liver was detected upon 24 h of cold exposure, but this did not translate into a potentiated rate of NAD^+^ synthesis (Figure 2D,E).

**Figure 7:**
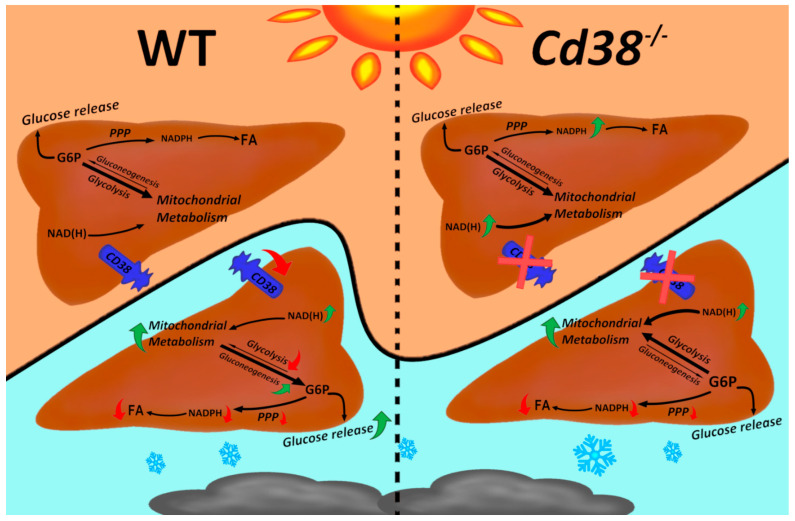
Scheme of cold-induced changes in liver from WT and *Cd38*^−/−^ mice. During cold exposure, CD38 expression is downregulated, and NAD(H) levels are increased. The pentose phosphate pathway (PPP) is downregulated, and NADPH levels are decreased, both in WT and *Cd38*^−/−^ mice, in agreement with a reduced fatty acid (FA) synthesis. The glycolytic pathway is also downregulated in WT mice, re-directing glucose-6-P (G6P) towards glucose release. Conversely, glycolysis is not downregulated in *Cd38*^−/−^ mice, indicating that CD38 is important for the glycolysis/gluconeogenesis cross-regulation. Sirtuin 3 activity, controlling mitochondrial metabolism, is enhanced by cold exposure in liver of WT mice, whereas it is upregulated in *Cd38*^−/−^ mice, regardless of the temperature.

A few studies have investigated the effect of cold acclimation on the expression of enzymes involved in glucose and lipid metabolism, yielding different and sometimes contradictory regulations. Here, we reported a marked influence of cold exposure in the regulation of both glucose and lipid metabolism in the liver. Figure 7 shows a scheme representing how cold exposure affects the major metabolic pathways in the liver of WT and *Cd38*^−/−^ mice.

Our data confirm that glucose oxidation through the pentose phosphate cycle is downregulated in the liver of WT mice during thermogenesis (Figure 3C,D). Additionally, glucose oxidation through glycolysis appears to be reduced in WT mice exposed to the cold, as indicated by the downregulation of PFK1 and GAPDH expression (Figure 4A–C) and by the decreased LDH enzymatic activity (Figure 4E). On the contrary, gluconeogenesis/glycogenolysis seems to be switched on: *G6Pase* expression and enzymatic activity rise during thermogenesis (Figure 4F,G), likely to release glucose and sustain metabolic pathways in the peripheral tissues. In line with this, we observed reduced hepatic G6P levels during thermogenesis in WT mice (Figure 4H), as well as reduced glycogen levels (Figure 4I).

The first difference observed in the liver between WT and *Cd38*^−/−^ mice is that the glycolytic pathway was not modified by cold acclimation in *Cd38*^−/−^ mice. As a matter of fact, no Pfk1, Gapdh, and LDH down-modulation in the liver of cold-exposed *Cd38*^−/−^ mice was observed (Figure 4A,B,D,E), which was different from WT mice. Furthermore, *Cd38*^−/−^ mice did not exhibit the induction of G6Pase (Figure 4F,G). In addition, we observed higher glycogen levels in the liver from *Cd38*^−/−^ mice in comparison with WT mice at 30 °C (Figure 4I), in line with the decreased expression of the hepatic glycogen phosphorylase observed in *Cd38*^−/−^ mice [38]. The higher glycogen levels in *Cd38* knockout mice possibly act as a broader glucose reservoir to promote glycolysis when energy and heat production are required, such as during cold exposure. Notwithstanding these differences, cold exposure caused glycogen consumption and G6P hydrolysis in *Cd38*^−/−^ mice, as well as in WT mice (Figure 4H,I).

Taken together, our data suggest a role for CD38 in regulating glycolysis and gluconeogenesis in the liver during thermogenesis. In line with our observation, the pharmacological inhibition of CD38 by the use of compound 78c suppressed gluconeogenesis in the liver [22]. In addition, previous studies investigated the effect of CD38 absence on glucagon-stimulated hepatic cells [37]: a link between the NAD^+^-derived, CD38-mediated Ca^2+^ signaling and glucagon-induced metabolic redirection towards gluconeogenesis was demonstrated. Specifically, glucagon treatment induced the expression of enzymes involved in gluconeogenesis, whereas CD38 inhibition or genetic depletion decreased hepatic cell sensitivity to glucagon: the expression levels of the *Pck1* gene (encoded for the gluconeogenic enzyme phosphoenolpyruvate carboxykinase 1) were not modified in glucagon-stimulated *Cd38*^−/−^ hepatocytes [37]. This result is in line with our data which shows the lack of stimulation of G6Pase upon cold exposure in *Cd38*^−/−^ mice (Figure 4F,G), and it may also be in agreement with the lower levels of glycerol observed in cold-stimulated *Cd38*^−/−^ mice (Figure 6D). Indeed, glycerol can be produced through glyceroneogenesis using the first steps of the gluconeogenic system. In addition, *Cd38*^−/−^ mice-derived hepatocytes displayed higher basal glycogen levels [37], in line with our data (Figure 4I). Overall, Rah and coll. ascribed the effects of the lack of CD38 to the impaired cADPR-dependent Ca^2+^ signaling [37]. As mentioned in the introduction, CD38 also catalyzes the synthesis of ADPR and 2dADPR, which are agonists of the transient receptor potential melastatin 2 (TRPM2), a ligand-gated Ca^2+^-permeable nonselective cation channel: the role of TRPM2 during cold exposure has still to be explored. Likewise, the role of NAADP^+^ in the liver during thermogenesis has not been reported. NAADP^+^ is a potent Ca^2+^-mobilizing second messenger whose synthesis can be mediated by CD38 starting from NADP^+^ in hepatocytes and in other cell types [41,42].

Besides the impaired cADPR-dependent Ca^2+^ signaling, at least some metabolic alterations observed in *Cd38*^−/−^ mice may also be due to the strong impact of CD38 deficiency on the NAD(P)(H) pool (Figure 1). For instance, the NAD^+^/NADH ratio regulates glycolysis and gluconeogenesis, with a low NAD^+^/NADH ratio being associated with enhanced gluconeogenesis [43,44]. In our condition, the NAD^+^/NADH ratio decreases in WT mice during cold exposure, in line with a fostered gluconeogenesis. Instead, the NAD^+^/NADH ratios is not modified during thermogenesis in *Cd38*^−/−^ mice, which is higher than in the cold-exposed WT mice. Thus, the different NAD^+^/NADH ratio in WT and *Cd38*^−/−^ mice might also be responsible for the altered regulation of glucose metabolism in hepatocytes, together with the modified calcium signaling, during cold/glucagon stimulation.

Regarding lipid metabolism, we observed increased hepatic TGs upon cold stimulation (Figure 6C), but also a downregulation of *Fasn* gene expression (Figure 6B) occurring in WT mice, confirming previous observations [40,45]. Similar modulation of TGs, *Fasn*, and *Acaca* occurred in *Cd38*^−/−^ mice upon cold acclimation (Figure 6A–C). At warm temperatures, *Cd38*^−/−^ seems to be able to synthetize higher levels of FA and release TGs since their levels are reduced in the liver and increased in the serum (Figure 6A–C,E).

The level of SOD acetylation, with _ac_SOD/SOD_tot_ being regarded as a marker of SIRT3 activity, was decreased in WT mice, demonstrating that the activity of SIRT3, a sirtuin with mitochondrial localization, is enhanced upon cold exposure (Figure 5B). This cold-mediated SIRT3 activation has been previously reported to occur in BAT [39]. Being SIRT3 a well-known fatty acid oxidation promoter, its activation suggests that higher hepatic TG levels observed in cold-exposed WT mice are not due to a slower β-oxidation. In fact, the higher hepatic TGs upon cold exposure may be due to an accelerated FA uptake [45,46].

In addition, our findings indicate that *Cd38*^−/−^ mice have higher hepatic SIRT3 activities in the basal conditions in comparison with WT mice (Figure 5D), but cold exposure has no effect in modulating this activity (Figure 5C); in other words, cold stimulation *per se* has no effect on SIRT3 activity in the liver of *Cd38*^−/−^ mice, in which NAD^+^ levels are very high (Figure 1A). This finding may suggest that the availability of the substrate NAD^+^ is one of the main regulators of SIRT3 activity. The impact of *Cd38* genetic ablation on SIRT3 regulation has been already investigated, although in an age-related context and not in a thermogenic-stimulating experimental setting [38]. The absence of CD38 enhanced SIRT3 and the total enzymatic activity. In addition, upon boosting NAD^+^ levels (obtained with exogenous NAD^+^ supplementation), SIRT3 activity increased to levels similar to those observed in *Cd38*^−/−^ mice which, on the other hand, already displayed high SIRT3 activity that could not be further enhanced [38]. Overall, these data are in line with our results, demonstrating that: (i) cold exposure increases NAD^+^ levels and hepatic SIRT3 enzymatic activity (Figure 1A and Figure 5B); (ii) the basal SIRT3 activity in the liver of *Cd38*^−/−^ mice is comparable to that observed in cold-stimulated WT mice (Figure 5C,D); (iii) the cold stimulus has no effect on the SIRT3 activity in *Cd38*^−/−^ mice (Figure 5C).

The NAD(H) pool, NADP^+^, NADPH, and the main enzymes involved in their metabolism were measured in the liver. Differently from previous results in WAT [29], NADP^+^ levels were unchanged in the liver of both WT and *Cd38*^−/−^ mice exposed to the cold (Figure 1C). Despite the over-expression of *Nadk* mRNA, NADK enzymatic activity was only slightly (not significantly) increased by cold exposure (Figure 3A,B), suggesting that post-transcriptional or post-translational regulations may prevent NAD^+^ redirection to produce NADP^+^ in this specific condition when NAD^+^ is necessary for SIRTs activity.

NADPH, the reduced NADP^+^ form, allows ROS-removing processes and lipid synthesis to occur. High mitochondrial activity may produce a large amount of ROS, which must be metabolized to avoid excessive intracellular stress. In line with this, NADPH levels were decreased in the liver of WT mice (Figure 1D). Moreover, as the expression of enzymes involved in lipid synthesis was repressed upon cold stimulation (Figure 6A,B), it is unlikely that the NADPH decrease reflects the NADPH utilization for lipid production. NADPH levels were higher in *Cd38*^−/−^ mice in comparison with WT mice at all temperatures (Figure 1D). However, cold exposure also determined an NADPH decline in *Cd38*^−/−^ mice, reflecting the same trend observed in WT mice (Figure 1D).

Cold exposure negatively regulated the levels of the two major NADP^+^-reducing enzymes, G6PD and the malic enzyme (Figure 3C–E), in line with the lower NADPH observed in WT and *Cd38*^−/−^ mice (Figure 1D). In light of these results, we hypothesized that G6PD expression was downregulated to avoid the diversion of G6P to the pentose phosphate pathway when it must be instead dephosphorylated and used to release glucose and lipid synthesis, which is not necessary. Curiously, the use of NADPH to counteract ROS production during cold temperatures appears not to be sustained by a stronger NADP^+^ reduction to NADPH. However, the present study was performed by exposing the mice to the cold for a rather short time: the influence of a long cold exposure needs to be investigated to unveil whether NADPH-producing mechanisms are promoted after 24 h of cold stimulation.

In WAT undergoing beiging, G6PD expression is regulated differently than in the liver: G6PD was increased in WAT and was likely to sustain the promoted lipogenesis induced by cold exposure when the NADPH demand rose. To the best of our knowledge, this is the first study reporting the effect of cold exposure in the liver of the NADP(H) pool, unveiling a different metabolic modulation in different organs during thermogenic stimulation.

CD38 seems to also affect the levels of FGF21 secreted by the liver (Figure 2G), which could promote adipose tissue thermogenic activation in an indirect manner [7]. Hence, hepatic CD38 may indirectly influence the metabolism in other peripheral tissues. Interestingly, the FGF21 release was recently linked to NAMPT overexpression, sequentially causing NAD^+^ increase, SIRT1 activation, and FGF21 production in the liver [35]. Likewise, high NAD^+^ levels in *Cd38*^−/−^ mice (Figure 1A) may determine FGF21 production (Figure 2G) through SIRT1 activation. On the other hand, hepatic NAMPT has been reported to exert both SIRT1-dependent and independent transcriptional regulations. Indeed, NAMPT levels were increased upon cold stimulation and in *Cd38*^−/−^ mice (Figure 2D,E). The increased FGF21, together with the increased BAs release (Figure 6F), is in line with the enhanced WAT browning and BAT activation observed in *Cd38*^−/−^ mice [29,30].

The relevance of the present study can also be seen from the perspective of NAD^+^ as fundamental in the regulation of energy metabolism: NAD^+^ decreases during aging, obesity, and obesity-related cardiometabolic diseases, in different tissues/organs, including the liver [20,23,24,25]. Thus, unveiling the different mechanisms affecting NAD^+^ homeostasis and its impact on metabolism is essential to address the approaches aiming to abate aging and dysfunctional metabolism. Indeed, several strategies have been proposed aiming to boost intracellular NAD^+^ and thereby promote healthy aging [9,47]. Increasing intracellular and NAD^+^ levels by the systemic administration of NAD^+^ biosynthetic precursors, such as nicotinamide riboside (NR) and its reduced form dihydronicotinamide riboside (NRH) [48,49], NMN and its reduced form NMNH [50], is one of the most promising approaches that has been extensively investigated in several pre-clinical studies and in human clinical trials [51,52].

CD38 inhibition is an alternative approach that has been investigated to boost NAD^+^ levels: our study demonstrates that CD38 reduction/absence does not impair and possibly even potentiates thermogenesis and is not detrimental to hepatic functions.

The present results show an important role of hepatic CD38 in regulating metabolic variations triggered by thermogenic stimulation and globally affecting systemic patterns of carbohydrate and lipid metabolism. Further studies are required to explore other ways of thermogenic stimulation, e.g., the diet- and drug-induced sympathetic activation of BAT.

## Figures and Tables

**Table 1:** qPCR primers.

Target Mouse Gene	Accession Number	Sequence, 5′-3′
*Nadk*	NM_001159637	Forward 5′-CCAAGTCTCGGAGCCTGTC-3′ Reverse 5′-AAATGTTGTCACTGGGCACG-3′
*Nampt*	NM_021524	Forward 5′-AATGTCTCCTTCGGTTCTGGTG-3′ Reverse 5′-CCCGCTGGTGTCCTATGTAAAG-3′
*Cd38*	NM_007646	Forward 5′-GGTCCTGATCGCCTTGGTAGTAG-3′ Reverse 5′-ATCTCCTGGCAGTTCTGATCTCTC-3′
*Acaca*	NM_133360	Forward 5′-CACTGTGGCTTCTCCAGCA-3′ Reverse 5′-CACCGACGGATAGATCGCAT-3′
*Fasn*	NM_007988	Forward 5′-ATGGGTGTGGAAGTTCGTCAG-3′ Reverse 5′-AGTGTGCTCAGGTTCAGTTGG-3′
*Me1*	NM_008615	Forward 5′-GGACCCGCATCTCAACAAGG-3′ Reverse 5′-AGGGCGGCAACAATCCATGA-3′
*G6pdx*	NM_008062	Forward 5′-TGATCGAGAAAAGCCCCAGC-3′ Reverse 5′-GTGAGGGTTCACCCACTTGT-3′
*Gapdh*	GU214026	Forward 5′-CGTGCCGCCTGGAGAAACCTG-3′ Reverse 5′-TGGAAGAGTGGGAGTTGCTGTTGAAG-3′
*Pfk1*	NM_001163487	Forward 5′-AGTTGGTATCTTCACGGGCG-3′ Reverse 5′-CATAGACACGCTCTCCCACG-3′
*Pdha1*	NM_008810	Forward 5′-GATGGAGCTAAAGGCGGATCA-3′ Reverse 5′-TCCGTAGGGTTTATGCCAGC-3′
*G6pase*	NM_008061	Forward 5′-AGCCAAGAGATGGTGTGAGC-3′ Reverse 5′-TACATGCTGGAGTTGAGGGC-3′
*β* *-2 Microglobulin*	NM_009735	Forward 5′-CGGTCGCTTCAGTCGTCAG-3′ Reverse 5′-CAGTTCAGTATGTTCGGCTTCC-3′
*Ubiquitin*	NM_019639	Forward 5′-GACAGGCAAGACCATCAC-3′ Reverse 5′-TCTGAGGCGAAGGACTAAG-3′
*Tbp*	NM_013684	Forward 5′-GAAGCTGCGGTACAATTCCAG-3′ Reverse 5′-CCCCTTGTACCCTTCACCAAT-3′
*β* *-actin*	NM_007393	Forward 5′-GCGAGAAGATGACCCAGATC-3′ Reverse 5′-GGATAGCACAGCCTGGATAG-3′
*Fgf21*	NM_020013	Forward 5′-CACACCGCAGTCCAGAAAGT-3′ Reverse 5′-CCTAGAGGCTTTGACACCCA-3′
